# The future of nursing education

**Published:** 2013-03-31

**Authors:** Jacqueline Dias

**Affiliations:** Aga Khan University, Karachi Pakistan

As I reflect on the last twenty years as a nurse educator I have witnessed the nursing education movement taking a number of sharp twists and turns and at the same time making inroads as well as finding its rightful place among other health care professionals.

From an apprenticeship model at the time of Florence Nightingale, nursing has moved out of hospitals and today has taken a university-based education approach. In addition, regulatory bodies have called for the baccalaureate degree as entry to practice for the profession of nursing beginning in the year 2020. The role of the nurse has expanded from that of bedside nurse and handmaiden of the physician coordinator of care in the community, to researcher, to clinical nurse specialist, to manager, leader and nurse practitioner within the health care system. These new emerging roles call for higher education and are determined by emerging health care needs such as palliative care, and changing demographics due to aging of the population and diversity of race and culture.

The writer believes that the impetus for nursing education to be heralded into the 21^st^ century is contingent upon a more educated work force with the minimum of a baccalaureate education and a move toward master’s and doctoral degrees. Attainment of higher education can no longer take place in the traditional way of sitting in a classroom. Nor can prospective students afford the luxury of taking time off from work to undertake a period of study. Flexible learning options, including part time study, accelerated programs, bridge programs and work based learning, are needed to ensure that nursing education is packaged and delivered in the most cost-effective and time-efficient way. Therefore, these aspects have to be built into the programs through the delivery of educational technologies such as e-Learning and distance learning. The future of nursing education necessitates a concurrent need for a change in the modes of assessment, introduction of new learning technologies, and educational strategies.

According to the literature, at present there is a heavy reliance on the Tylerian model in nursing. This means that the approach to curriculum development is routed in behavioral objectives and evaluation methods to assess if each objective is met or not. Students complain that they are recipients of experiences selected by the nurse teachers and curriculum committees. Nurse educators will have to respond differently and teach the curriculum differently. In order to prepare nurses with new and different perspectives and abilities who can function well in a rapidly changing environment, a new paradigm would need to emerge. Tanner 1990 has referred to this as the time when “nurse educators tacitly or explicitly agree to a new world view of educational practices.”^1^ The new paradigm will be reflective of major social changes, and the arising needs and transformation of healthcare. More emphasis will be on a range of collaborative activities, mechanisms, and structures which have arisen as likely options in recognition of the interdependence of service and education sectors.

In order to run these flexible kinds of programs there must be adequate faculty preparation along with faculty practice to keep abreast of changes in the field of nursing. This will become the driving force to encourage new approaches to teaching and learning based on a philosophy consistent with modern demands and modern teaching pedagogies and flexible learning programs.

Furthermore, the future of nursing education programs depends largely on available resources. This includes the use of skills training and simulations in a controlled setting.

Teaching pedagogy will need to include a hybrid of strategies. Hybrid design is a mix of traditional classroom and online teaching methods. Creation of learning environments through technology allows for flexible course delivery. E learning methodologies allow for flexibility because students have access to course materials 24 hours a day 7 days a week. In this way students would be able to manage their academic responsibilities and home environments.

## Conclusion

In order to transform the face of nursing education for the 21^st^ century, nurses would be equipped with the competencies and skills mix to take care of diverse population, changing demographics and emerging health needs. Therefore, the future of nursing education must spell out competencies in health care financing, community and public health, leadership, quality patient centered care, and a commitment to research and evidence based practice. Only then will future nurses go from being citizens of a country to denizens of the world.
